# Clinical features of hereditary angioedema involving the gastrointestinal tract: A retrospective analysis^☆^

**DOI:** 10.1016/j.waojou.2026.101252

**Published:** 2026-01-31

**Authors:** Haiyuan Ma, Danping Zheng, Yangdi Wang, Chunyang Tian, Zhoulin Huang, Shanshan Xiong, Yangyang Ke, Ren Mao, Yao He, Minhu Chen, Xuehua Li, Baili Chen

**Affiliations:** a Department of Gastroenterology, The First Affiliated Hospital, Sun Yat-sen University, Guangzhou, China; b Department of Radiology, The First Affiliated Hospital, Sun Yat-sen University, Guangzhou, China; c Clinical Medicine, Sun Yat-sen University, Guangzhou, China; d Department of Gastroenterology, The First Affiliated Hospital, Sun Yat-sen University, Guangzhou, 510080, China; e Department of Radiology, The First Affiliated Hospital, Sun Yat-sen University, Guangzhou, 510080, China

**Keywords:** Hereditary angioedema, Gastrointestinal edema, Clinical features, Radiological features, Treatment

## Abstract

**Background:**

Hereditary angioedema (HAE) is a rare, severe, disabling, and life-threatening disorder characterized by recurrent and unpredictable edema of skin and mucous membranes. Gastrointestinal edema, often presenting as abdominal pain, is common but frequently misdiagnosed, leading to unnecessary surgeries. This study aims to characterize gastrointestinal edema in HAE patients to improve early diagnosis and guide treatment.

**Method:**

We analyzed 61 patients with HAE who were admitted in our hospital between April 1, 2023 and August 31, 2025. Data on demographic characteristics, clinical manifestations, laboratory findings, imaging results, and treatment outcomes were collected through questionnaires and hospital electronic records.

**Result:**

Among the patients, 86.9% experienced gastrointestinal edema, mostly characterized by abdominal pain (94.3%), nausea (79.2%), and diarrhea (71.7%). Misdiagnosis occurred in 73.6% of cases, and 22.6% underwent unnecessary surgeries. The median age of gastrointestinal edema onset was 16.0 years, with the most severe episodes occurring at a median age of 25.0 years. The median annual frequency of attacks was 4.0/year, with a median severity score of 7.0. Acute attacks showed elevated D-dimer, white blood cells, neutrophils, and hemoglobin. CT imaging frequently revealed intestinal wall thickening and abdominal-pelvic effusion. Lanadelumab demonstrated superior efficacy over danazol in reducing both the frequency and severity of edema attacks, while icatibant significantly shortened the duration of edema episodes.

**Conclusion:**

This study updates the clinical and imaging features of gastrointestinal edema in Chinese patients with HAE, identifies biomarkers of gastrointestinal edema attacks, and highlights the efficacy of lanadelumab and icatibant, aiding timely diagnosis and treatment.

## Introduction

Hereditary angioedema (HAE) is a severe, disabling, life-threatening autosomal dominant disorder characterized by recurrent and unpredictable skin and mucosal edema,^1^^,^^2^ with an estimated global prevalence of 1 in 50,000.^3^ In 1963, Donaldson et al found that deficiency of the C1 esterase inhibitor (C1–INH) encoded by the *SERPING1* gene is a genetic feature of HAE.^4^ According to the mutant gene and the level of C1–INH, HAE can be broadly divided into 2 main types: HAE with C1 inhibitor deficiency (HAE-C1-INH) and HAE with normal C1 inhibitor levels (HAE-nC1-INH).^5^^,^^6^ The HAE-C1-INH can be further divided into 2 subtypes: hereditary angioedema type I (HAE-I), with low antigenic level and function of C1 inhibitor, and hereditary angioedema type II (HAE-II) with normal or elevated antigenic level but low function of C1 inhibitor as a result of mutations affecting the reactive loop of the molecule.^7^, ^8^, ^9^ The clinical characteristics of HAE are recurrent episodes of subcutaneous and submucosal edema, which can involve the skin, gastrointestinal tract, respiratory tract, etc.^10^ A recent international study showed that patients with HAE-I and HAE-II accounted for 85% and 15% of all HAE patients,^7^ respectively. In a Chinese cohort, HAE-I and HAE-II patients accounted for 96.3% and 3.7%, the proportions of skin edema, gastrointestinal edema, and laryngeal edema were 99.1%, 72.0%, and 60.7%, respectively.^11^ The main gastrointestinal manifestations of HAE are severe abdominal pain, nausea, vomiting, etc,^12^ which were often misdiagnosed as acute abdomen, especially when there is no edema elsewhere.^13^ Due to the lack of understanding of HAE by clinicians, the diagnosis is often delayed, and may lead to unnecessary surgical procedures.^14^ The misdiagnosis of gastrointestinal edema not only significantly reduces the quality of life of patients with HAE, but also increases the economic burden of patients due to repeated medical treatment.^15^ At present, data on clinical and imaging features of the gastrointestinal HAE remain scarce.

The purpose of this study was to analyze the clinical manifestations, laboratory characteristics, imaging features, and treatment outcomes in a Chinese cohort of HAE, with a particular focus on gastrointestinal edema.

## Methods

### Patients

A retrospective observational study was conducted in the Department of Gastroenterology of the First Affiliated Hospital of Sun Yat-sen University from April 1, 2023 to August 31, 2025. A total of 61 adult patients diagnosed with HAE were included in this study, including HAE-C1-INH (HAE-I and HAE-II) and HAE-nC1-INH. The diagnosis of HAE was based on medical history (recurrent angioedema, without urticaria, affecting the skin and/or gastrointestinal and/or larynx) and laboratory findings (HAE-I: repeatedly confirmed low plasma C4 and C1–INH levels; HAE-II: normal or elevated C1–INH levels but functional decline>50% normal value; HAE-nC1-INH: confirmed by genetic testing).^3^ The research protocol was approved by the Research and Ethics Committee of the First Affiliated Hospital of Sun Yat-sen University. Participants were informed about the purpose of the survey, the institution conducting the study, and the privacy protections. Informed consent has been obtained from all patients prior to the study, and patients have signed informed consent forms for the release of their data.

### Demographic and clinical data collection

Demographic data and clinical information regarding the location, severity, triggers, duration of acute HAE attack, misdiagnosis and response to treatment were collected from electronic medical records and a specific questionnaire [Supplementary file]. All participants completed the questionnaires, and any incomplete or ambiguous feedback was reconfirmed.

### Laboratory data collection

Hematological, inflammatory, biochemical, and coagulation markers, as well as C1-inhibitor levels, complement C4 levels, and other relevant indicators, from 15 patients during edema attacks and remission periods, were obtained from the outpatient, emergency and inpatient departments [Supplementary file].

### Imaging data collection

Acute gastrointestinal edema imaging during attacks of HAE was collected from 9 patients through the PACS system of the First Affiliated Hospital of Sun Yat-sen University, using Dual-layer Spectral Detector CT (DLCT).

### Statistical analysis

Descriptive statistics were reported as counts, percentages, means with standard deviations, and medians with interquartile ranges (IQR). For comparisons between the 4 independent predominant-site groups (gastrointestinal-predominant, skin-predominant, mixed-type, and laryngeal-predominant), the Kruskal-Wallis test was used for non-normally distributed variables, and one-way ANOVA was used for normally distributed variables. When significant differences were detected, post-hoc multiple-comparison tests were performed (Dunn's test after Kruskal-Wallis; Tukey's HSD after ANOVA). The site-specific analysis (skin, gastrointestinal, and laryngeal episodes) was conducted descriptively, as the same patient could contribute data to multiple anatomical sites; therefore, no statistical comparisons were performed for this part of the analysis. For the analysis of serum biomarkers and pairwise subgroup comparisons, the independent-samples *t*-test was used for normally distributed variables, while the Mann-Whitney *U* test was used for non-normally distributed variables. A two-sided p-value <0.05 was considered statistically significant, statistical significance was denoted as follows: ∗p < 0.05, ∗∗p < 0.01, and ∗∗∗p < 0.001. All analyses were conducted using GraphPad Prism 9 (GraphPad Software, San Diego, CA, USA).

## Results

### Comparison of gastrointestinal, skin and laryngeal edema

We first characterized the clinical features of edema using a site-specific descriptive analysis of all 61 patients, acknowledging that individuals with HAE may experience edema in multiple anatomical sites, either simultaneously during the same episode or sequentially across different attacks. Among all patients, 53 had experienced gastrointestinal edema, 59 had experienced skin edema, and 33 had experienced laryngeal edema. The median age of onset was 16.0 years (IQR = 18.0) for gastrointestinal edema, 17.0 years (IQR = 15.0) for skin edema, and 30.0 years (IQR = 14.0) for laryngeal edema. The median age at which the most severe edema occurred was 25.0 years (IQR = 17.0) for both the gastrointestinal tract and skin, and 30.0 years (IQR = 16.0) for the larynx. Before any treatment, the median annual frequency of episodes was 4.0 per year (IQR = 8.0) for gastrointestinal edema, 4.0 per year (IQR = 8.0) for skin edema, and 1.0 per year (IQR = 1.0) for laryngeal edema. The median severity scores were 7.0 (IQR = 4.5), 6.0 (IQR = 5.0), and 6.0 (IQR = 5.5) for gastrointestinal, skin, and laryngeal episodes, respectively.

**Fig. 1:**
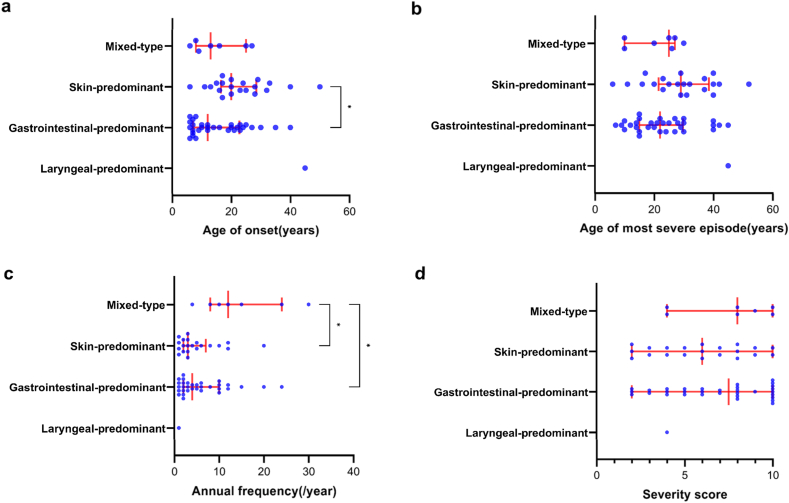
Age of onset, age at onset of most severe episode, annual frequency, and severity score of edema attacks of different patient classification (Mixed-type: n = 7, skin-predominant: n = 21, gastrointestinal-predominant: n = 32, laryngeal-predominant: n = 1).

To enable group-level comparisons, we also categorized patients into 4 mutually exclusive groups according to their predominant edema site since the onset of their disease, as reported in the questionnaire. Gastrointestinal-predominant patients constituted the largest proportion (n = 32, 52.5%), followed by skin-predominant patients (n = 21, 34.4%), mixed-type patients (n = 7, 11.5%), and laryngeal-predominant patients (n = 1, 1.6%). Gastrointestinal-predominant patients had an earlier median age of edema onset (12.0 years, IQR 15.8) compared with skin-predominant patients (20.0 years, IQR 12.0; p = 0.0493) (Fig. 1a). The median age of the most severe episode did not significantly differ among groups, although gastrointestinal-predominant (22.0 years, IQR 14.8) and mixed-type patients (25.0 years, IQR 17.0) showed numerically earlier ages compared with skin-predominant patients (29.0 years, IQR 17.0) (Fig. 1b). The mixed-type group demonstrated the highest annual attack frequency (12.0/year, IQR 16.0), significantly greater than both the skin-predominant (3.0/year, IQR 5.0; p = 0.0466) and gastrointestinal-predominant groups (4.0/year, IQR 8.0; p = 0.0255) (Fig. 1c). No significant differences in attack severity score were observed across the 4 predominant-site groups (Fig. 1d). Interpretation of differences involving the laryngeal-predominant group is limited by the very small sample size (n = 1).

### Clinical manifestations of gastrointestinal edema

**Fig. 2:**
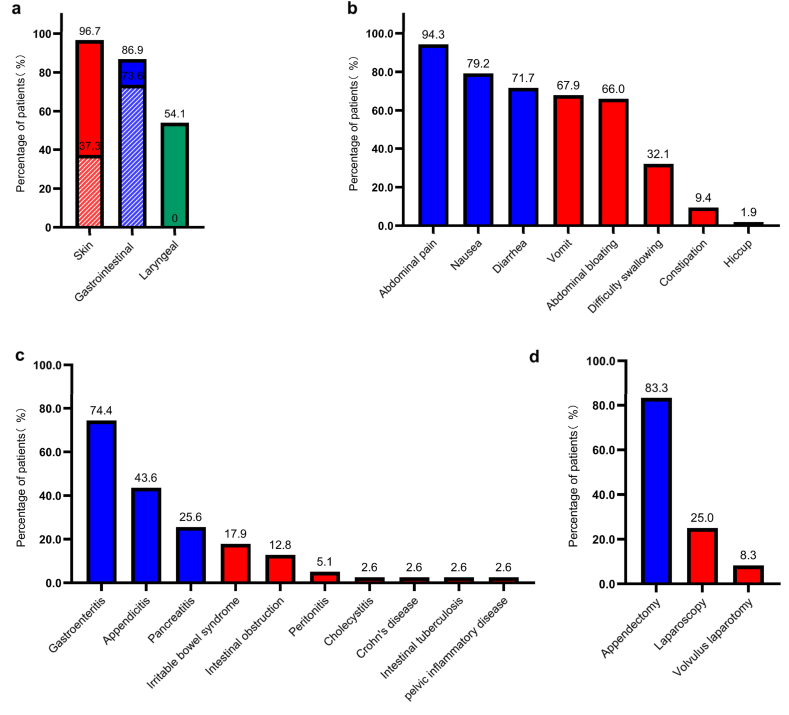
Clinical manifestations of hereditary angioedema with gastrointestinal edema. (a) Percentage of patients with edema at different sites (n=61), including the percentage of different locations as the first attack site. (b) Percentage of patients with different gastrointestinal edema symptoms (n=53). (c) Misdiagnosis rate of patients with gastrointestinal edema (n=39). (d) Percentage of patients with gastrointestinal edema who underwent unnecessary surgeries due to misdiagnosis (n=12).

In our survey, 86.9% (53/61) of the patients with HAE experienced gastrointestinal edema, and 73.6% (39/53) of the patients had gastrointestinal edema as the first attack site, which was higher than the proportion of skin edema as the first attack site (37.3%, 22/59) (p = 0.0001) (Fig. 2a). Among the 53 patients who had gastrointestinal edema of HAE, with a mean age of 37.9 years, 73.6% (39/53) patients were HAE-I, and 18.9% (10/53) patients were diagnosed with HAE-II. About one third of patients (32.1%, 17/53) were prone to drug or food allergies, and 86.8% (46/53) of patients have a family history of the disease (Table 1). There were 58.5% (31/53) of patients who had comorbidities, including metabolic diseases, joint swelling and pain, recurrent oral ulcers [Supplementary table. 1]. Among all 53 gastrointestinal patients, the median age of onset was 13.0 years (7.5, 21.5), the median duration of the disease was 20.0 years (7.0, 29.0), and the median time from the first symptoms to diagnosis was 18.0 years (7.0, 29.0). The symptoms of gastrointestinal edema include abdominal pain (94.3%), nausea (79.2%), diarrhea (71.7%), vomiting (67.9%), abdominal bloating (66.0%), difficulty in swallowing (32.1%), constipation (9.4%) and hiccup (1.9%) (Fig. 2b). The main triggers of gastrointestinal edema attacks were physical exhaustion (50.9%), mood swings (50.9%), and weather changes (39.6%) [Supplementary table. 2]. When gastrointestinal edema attacks occurred, 18.9% (10/53) of patients experience prodromal symptoms, and 5 patients presented with abdominal erythema, 2 with thirst, 1 with increased bowel sounds, 1 with irritability, and 1 with fatigue. In addition, 39 patients (73.6%) were misdiagnosed as gastroenteritis (74.4%), appendicitis (43.6%), pancreatitis (25.6%), irritable bowel syndrome (17.9%), intestinal obstruction (12.8%), peritonitis (5.1%), cholecystitis (2.6%), Crohn's disease (2.6%), intestinal tuberculosis (2.6%), or pelvic inflammatory disease (2.6%) (Fig. 2c). Twelve patients (22.6%) with gastrointestinal edema underwent unnecessary surgical procedures, including appendectomy (83.3%), laparoscopic exploration (25.0%), and even volvulus laparotomy (Fig. 2d).

**Table 1:** The demographic features of gastrointestinal edema (n = 53)

Demographics of patients with gastrointestinal edema in HAE (N = 53)	Mean ± SD or Median (Q1,Q3) or n (%)
Sex	
Female	32 (60.4)
Male	21 (39.6)
Age (years)	37.9 ± 16.3
BMI(Kg/㎡)	22.5 ± 3.5
Type	
HAE-1	39 (73.6)
HAE-2	10 (18.9)
HAE-nC1-INH	4 (7.5)
Smoking history	14 (26.4)
Alcohol history	21 (39.6)
Allergy history	17 (32.1)
Family history	46 (86.8)
Comorbidity	31 (58.5)
Age of onset (years)	13.0 (7.5,21.5)
Duration of disease (in years)	20.0 (7.0,29.0)
Delayed diagnosis time (in years)	18.0 (7.0,29.0)

### Laboratory examinations

**Fig. 3:**
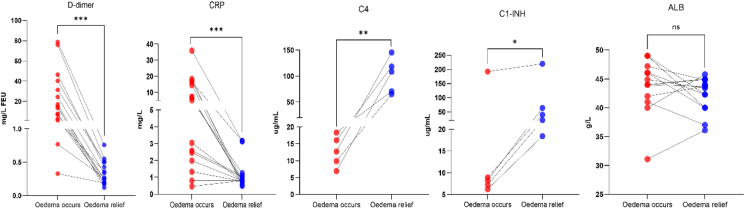
Comparison of different reactant levels between the phases of edema attacks and remission (n = 15).

**Fig. 4:**
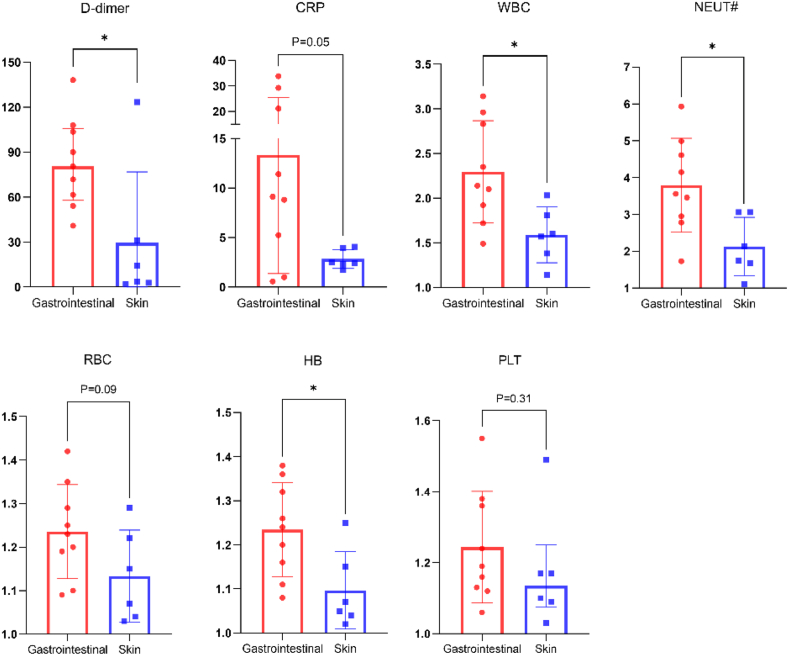
Comparison of the fold changes in reactants between gastrointestinal edema (n = 9) and skin edema (n = 6).

Peripheral blood samples were collected from 15 patients during edema attacks and remission periods. It was observed that serum C-reactive protein, white blood cell count, neutrophil count, red blood cell count, hemoglobin, platelet count levels were significantly lower during remission compared to the attack period in all patients [Supplementary Fig. 1]. Interestingly, D-dimer, a marker for coagulation and fibrinolysis, and hypothesized to contribute to an increased risk of VTE,^16^ was significantly increased in the attack phase and reduced in the remission period (p < 0.0001). There was no significant change in albumin levels between the 2 phases. Also, consistent with the HAE management guidelines that recommend using complement C4 as an initial screening marker for HAE,^3^ both C1–INH and C4 levels were significantly higher during remission than the attack period in all 15 patients (Fig. 3). The above edema attacks included 9 episodes of gastrointestinal edema and 6 episodes of skin edema. By comparing the fold changes in reactant levels between edema attacks and remission in different sites, we found that D-dimer is significantly elevated during the attack phase of gastrointestinal edema than that of skin edema (p = 0.03). Other laboratory markers, including white blood cell count, neutrophil count, hemoglobin, were significantly higher in the attack phases of gastrointestinal edema compared to skin edema (Fig. 4).

### Imaging features of gastrointestinal edema

**Fig. 5:**
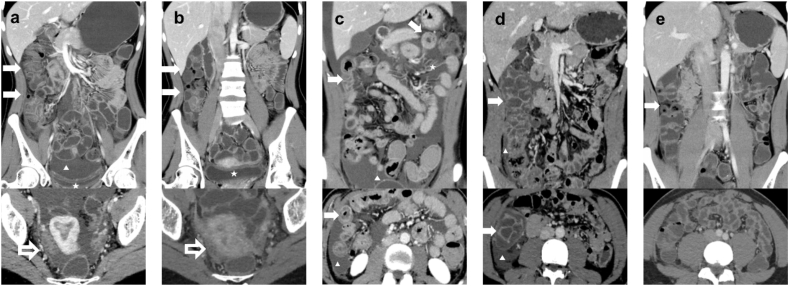
Abdominal CT images of gastrointestinal edema onset and remission in 2 patients. (a-b) This is a 26-year-old female patient who presented with a one-day history of abdominal pain and subsequently underwent both non-contrast and contrast-enhanced CT imaging. The upper image of fig. 5a demonstrates edema and thickening of the right colon along (→) with pelvic effusion (▲), as well as edema of the bladder wall (★). The lower image of fig. 5a reveals mild ovarian swelling (→). Following admission, the patient's abdominal pain resolved after receiving icatibant medication. A follow-up CT (Fig. 5b) examination conducted the next day indicated that the thickening of the right colon has largely subsided, ascites is fully absorbed, there is significant improvement in bladder wall edema (★, upper image of fig. 5b); and the lower image of fig. 5b confirmed resolution of bilateral ovarian swelling (→). (c-e) A 21-year-old male patient presented to our hospital for the first time with complaints of abdominal pain. Initial coronal and axial CT imaging (Fig. 5c) revealed edema and thickening of the small intestine in the mid-to-upper abdomen (→), characterized by target sign, along with surrounding ascites (▲) and comb sign features (★). One month later, the patient was readmitted due to recurrent abdominal pain. CT imaging (Fig. 5d) demonstrated significant edema and thickening of the right colon (→), with more pronounced target sign, and a small amount of ascites (▲) was noted. Upon admission, icatibant therapy was initiated. A subsequent CT conducted 1.5 days later (Fig. 5e) indicated complete resolution of all previous abnormal imaging findings, coinciding with the alleviation of abdominal pain symptoms.

Nine patients received abdominal and pelvic CT scans, including non-contrast and contrast-enhanced imaging, when they experienced abdominal pain. All showed varying degrees of abdominal or pelvic effusion. In 8 out of 9 patients, continuous or segmental submucosal edema caused notable thickening of the intestinal wall, averaging 10.0 mm. This thickening showed characteristic “target signs” on both non-contrast and contrast-enhanced imaging. In our study, we also identified comb signs surrounding the affected intestine in 4 patients and observed continuous submucosal fat deposits in 3 patients (Table 2). Of the 9 patients, 2 underwent CT re-evaluation within 1–3 days following the alleviation of abdominal pain. The follow-up imaging revealed that the previously identified abnormal findings had resolved concurrent with the improvement in symptoms (Fig. 5).

**Table 2:** CT imaging features of gastrointestinal edema (n = 9)

CT imaging features (N = 9)	n (%) or Mean ± SD
The areas of gastrointestinal edema or thickening	8 (88.9)
Small intestine	2 (25.0)
Colorectum	1 (12.5)
Stomach + small intestine	1 (12.5)
Small intestine + Colorectum	2 (25.0)
Stomach + small intestine + Colorectum	2 (25.0)
Maximum thickness of the bowel wall (mm)	10.0 ± 4.8
Pattern of distribution	8 (88.9)
Segmental	3 (37.5)
Continuous	5 (62.5)
Target sign	8 (88.9)
Comb sign	4 (44.4)
Abdominal-pelvic effusion	9 (100.0)
Submucosal fat deposition in the bowel	3 (33.3)

### Treatment outcomes

**Fig. 6:**
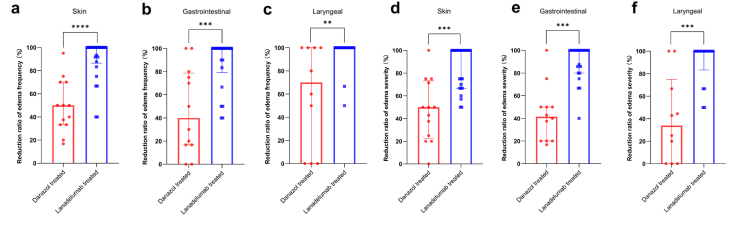
The therapeutic effects of lanadelumab and danazol (lanadelumab: n = 26, danazol: n = 13). (a) The reduction ratio in the frequency of skin edema attacks after treatment with danazol (n=13) and lanadelumab (n=26), compared to baseline. (b) The reduction ratio in the frequency of gastrointestinal edema attacks after treatment with danazol (n=12) and lanadelumab (n=22), compared to baseline. (c) The reduction ratio in the frequency of laryngeal edema attacks after treatment with danazol (n=10) and lanadelumab (n=17), compared to baseline. (d) The reduction ratio in the severity scores of skin edema attacks after treatment with danazol (n=13) and lanadelumab (n=26), compared to baseline. (e) The reduction ratio in the severity scores of gastrointestinal edema attacks after treatment with danazol (n=12) and lanadelumab (n=22), compared to baseline. (f) The reduction ratio in the severity scores of laryngeal edema attacks after treatment with danazol (n=10) and lanadelumab(n=17), compared to baseline.

**Fig. 7:**
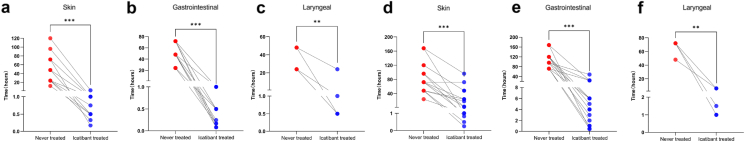
The therapeutic effects of icatibant for different edema sites (skin: n = 16, gastrointestinal: n = 18, larynx: n = 5). (a-c) The changes in the time to initial resolution of edema when treated with icatibant compared to that without any treatment. (d-f) The changes in the time to complete resolution of edema when treated with icatibant compared to that without any treatment.

Among all the above HAE patients, the preventive treatments included danazol and lanadelumab. Evaluation of the efficacy of these 2 medications was based on the frequency of edema attacks and severity scores at different sites, meaning the percentage decrease from baseline in these parameters after treatment. We found that lanadelumab was superior to danazol in both reducing the frequency of edema attacks and alleviating their severity (Fig. 6). Moreover, 46.2% (6/13) of danazol users experienced adverse reactions, such as acne, abnormal liver function, menstrual disorders, weight gain, etc, while 38.5% (10/26) of lanadelumab users experienced adverse reactions, primarily local injection site reactions. In terms of on-demand treatment, patients used icatibant for acute attacks. Thirteen patients used it during skin edema attacks, sixteen patients used it during gastrointestinal edema attacks, and 3 patients used it during laryngeal edema attacks. The use of icatibant for acute edema attacks at different sites significantly shortened the time to symptom relief and the time to complete symptom relief (Fig. 7). Our findings showed that icatibant has a significant advantage in shortening the duration of edema, and 40.0% (10/25) of icatibant users experienced adverse reactions, primarily local injection site reactions.

## Discussion

In our center, 96.7% of the 61 patients with HAE had skin edema, 86.9% had gastrointestinal symptoms, and 54.1% had laryngeal edema. The proportion of gastrointestinal involvement was higher than that reported by Peking Union Medical College Hospital,^11^ and similar to foreign reports,^17^ which may reflect that our department primarily enrolled patients seeking care for gastrointestinal symptoms. Of the 53 (86.9%) patients with HAE who had gastrointestinal edema, 39 had HAE-I, 10 had HAE-II, and 4 had HAE-nC1-INH. The higher proportion was found in HAE-II, which might be related to the higher number of families found in HAE-II. And 86.8% of patients had family history, which underscores the significance of family history in HAE diagnosis and advises clinicians to perform thorough family screening for every patient. Recent studies have shown that HAE patients are at a higher risk of autoimmune diseases and chronic comorbidities,^18^^,^^19^ our study also found that about one third of patients had allergy history, and 58.5% of patients had comorbidities, suggesting that while treating HAE, the management of comorbidities should not be overlooked.

The median age of onset of gastrointestinal symptoms in our center was 13.0 years old, the median duration of disease was 20.0 years, and the median delay from the first onset of symptoms to diagnosis was 18.0 years. This is longer than the situation reported by Peking Union Medical College Hospital^11^ and that of Western countries,^20^ suggesting that there were fewer opportunities for pediatric patients in China to visit the pediatric department for abdominal discomfort, as well as possibly due to current insufficient understanding of HAE in our medical staff, resulting in a large number of misdiagnosis and missed diagnosis. The main symptom of HAE patients with gastrointestinal involvement is abdominal pain, which is severe and can be relieved spontaneously. Thirty-nine cases (73.6%) had gastrointestinal symptoms at the time of the first episode, and 39 (73.6%) patients experienced misdiagnosis, even underwent unnecessary surgery, which was similar to that reported by Peking Union Medical College Hospital,^11^ and higher than that reported abroad.^13^ The high misdiagnosis rate reminds us to improve not only the diagnostic awareness of HAE, but also the ability to differentiate and diagnose abdominal pain.

C1 inhibitor (C1–INH), encoded by the *SERPING1* gene, serves as a critical negative regulator of multiple proteolytic pathways.^1^^,^^21^ It inhibits the C1 components of the classical complement pathway, MASP-1 and MASP-2 of the lectin pathway, and key factors of the contact pathway, including prekallikrein, FXIIa, and FXI^7^^,^^22^, ^23^, ^24^, ^25^, ^26^, ^27^, ^28^, ^29^. Deficiency of C1–INH results in hyperactivation of the contact pathway, which promotes bradykinin (BK) and thrombin generation.^30^^,^^31^ This dysregulation contributes to the increased vascular permeability, elevated systemic inflammatory biomarkers and coagulation activation markers during attacks of HAE, particularly D-dimer.^30^^,^^32^ D-dimers is considered a reliable and sensitive index of thrombosis in vivo and indicative of a dynamic process of thrombus formation and lysis.^33^ Clinically, elevated D-dimer levels are observed in various thrombotic conditions, including venous thromboembolism (VTE).^34^ A recent prospective case-control study in the general population also supports a role for plasma C1–INH in modulating VTE risk.^35^ Patients with elevated plasma levels of C1–INH were found to have a significantly lower risk for VTE than those with lower levels of C1–INH.^35^ Moreover, increasing plasma C1–INH levels were shown to significantly reduce thrombin generation initiated by the contact pathway.^35^ Notably, patients with HAE have been found to have an elevated risk of VTE.^16^^,^^36^ By comparing serum biomarkers during the onset and resolution of edema at different sites in fifteen patients, we confirmed a significant increase in D-dimer during acute attacks, particularly during episodes of gastrointestinal edema. These findings underscore the importance of monitoring for visceral thrombosis and the potential development of ischemic bowel disease during episodes of gastrointestinal edema.

The most observed intestinal CT characteristics in patients with HAE during flare-ups included intestinal wall thickening and abdominal-pelvic effusion.^37^ In this study, CT imaging was available for 9 patients with gastrointestinal edema. Among them, all exhibited abdominal or pelvic effusion, suggesting that this may be the most significant imaging characteristic for this patient population, though it has limited specificity in differentiating the disease. Eight of 9 patients (88.9%) showed intestinal wall thickening, attributed to pronounced submucosal edema. Specifically, this submucosal edema manifested as a characteristic target sign on CT images, defined by alternating layers of slightly increased, markedly decreased, and slightly increased CT density from the innermost to the outermost layer. Importantly, this target sign was evident not only on contrast-enhanced CT images but also on non-contrast images, owing to the substantial reduction in density caused by the pronounced submucosal edema. The bowl wall thickening can be continuous or segmental, and our study found both types to be equally prevalent. Additionally, in this small series, the jejunum is the most commonly affected site, followed by the colon and then the stomach. Moreover, in our study, 4 of 9 patients (44.4%) exhibited comb sign (engorged vasa recta). This sign is predominantly observed in patients with Crohn's disease; however, it may also be noted in patient with HAE, potentially indicating hyper-perfusion of the affected bowel. Additionally, we identified possible new finding of submucosal fat deposition in 33.3% (3/9) of patients, which has not been previously documented in HAE patients. Further investigation is needed to explore potential causative factors and gather additional patient imaging data for comprehensive analysis.

The treatment of HAE includes both preventive and on-demand treatments. The international WAO/EAACI guideline recommends any of the plasma-derived C1–INH, lanadelumab and berotralstat as the first-line long-term prophylactic treatment of patients with HAE-1/2, while icatibant, ecallantide and intravenous C1–INH are the recommended on-demand treatments of choice.^3^ In China, the current preventive medications available to patients include lanadelumab^38^ and danazol.^39^ We compare the efficacy of these 2 preventive treatments, confirming the superiority of lanadelumab. Also, as a specific and selective competitive antagonist of the bradykinin B2 receptor and prevents binding of bradykinin to its receptor,^40^ icatibant can significantly reduce the duration of edema and the severity of the disease. Therefore, in addition to emergency medication such as icatibant, patients with conditions should be treated prophylactically for frequent episodes or those with a history of laryngeal edema.

This study has several limitations that should be considered when interpreting the results. One major limitation is the relatively small sample size, which resulted in insufficient statistical power and may have affected the validity of the statistical analyses. Another limitation is the single-center design of this study. The results are based on data collected from a single institution, which limits the representativeness and generalizability of the findings. Additionally, selection bias should be acknowledged, leading to a higher reported prevalence of gastrointestinal edema compared to studies^11^^,^^41^ conducted in departments that serve a broader spectrum of HAE patients (eg, Department of Allergy). A further limitation is the retrospective design, which relies on participants' ability to recall past events or provide self-reported data. This introduces the potential for information bias and recall bias, which could affect the reliability of the findings. Therefore, future research should include larger sample sizes and multi-center prospective studies to enhance the credibility of the conclusions drawn from this study.

## Conclusion

This study updates the clinical manifestations and imaging features of gastrointestinal edema in Chinese patients with HAE. Clinical physicians are advised to focus on HAE screening in young patients presenting with abdominal pain and a positive family history. We identify the biomarkers that change significantly during gastrointestinal edema attacks, suggesting that the risk of visceral thrombosis and the potential concomitant development of ischemic bowel disease should be given attention during episodes of gastrointestinal edema. Lanadelumab show a greater advantage over danazol in preventive treatment, and icatibant can significantly reduce the duration of edema and the severity of the disease in on-demand treatment.

## Author contributions

All authors made substantial contributions to the conception, design, drafting and revision of this manuscript and gave final approval of the version to be published.

## Ethics statement

The research was approved by the Research and Ethics Committee of the First Affiliated Hospital of Sun Yat-sen University ( [2025] 170 ). Informed consent has been obtained from all patients prior to the study, and patients have signed informed consent forms for the release of their data.

## Author's consent

All authors agreed to publication of this work.

## Declaration of Generative AI and AI-assisted technologies in the writing process

Nothing to disclose.

## Funding

The authors acknowledge financial support from Guangdong Weiji Medical Development Foundation (K-20240204).

## Declaration of competing interest

There are no competing interests for any author.
