# Supplementary material for: Clinical features of hereditary angioedema involving the gastrointestinal tract: A retrospective analysis
Source: World Allergy Organ J. 2026 Jan 31;19(2):101252. doi: 10.1016/j.waojou.2026.101252 (PMC12886538; doi:10.1016/j.waojou.2026.101252)
Supplement: Multimedia component 1 [file mmc1.doc]

**Supplementary method**

**Laboratory data collection**

White blood cell count (WBC) (normal range: 4.0–10.0 × 10^9/L), neutrophil count (NEUT#) (normal range: 1.8–6.4 × 10^9/L), red blood cell count (RBC) (normal range: 4.0–5.5 × 10^12/L), hemoglobin (HB) (normal range: 130–175 g/L), platelet count (PLT) (normal range: 100–300 × 10^9/L) was measured by automated cell counter. Serum C-reactive protein (CRP) (normal range: 0–3 mg/L), and plasma D-dimer (normal range: 0–0.55 mg/L FEU) was measured by immunoturbidimetric assay. Serum albumin (ALB) (normal range: 35.0–50.0 g/L) was measured by bromocresol green method. C1-inhibitor concentration (C1-INH) (normal range: 81.46–291.29 ug/mL) and complement C4 concentration (C4) (normal range: 72.85–372.95 ug/mL) was obtained by tandem mass spectrometry.
