# Supplementary material for: Clinical features of hereditary angioedema involving the gastrointestinal tract: A retrospective analysis
Source: World Allergy Organ J. 2026 Jan 31;19(2):101252. doi: 10.1016/j.waojou.2026.101252 (PMC12886538; doi:10.1016/j.waojou.2026.101252)
Supplement: Multimedia component 2 [file mmc2.doc]

HAE Survey Questionnaire of the First Affiliated Hospital of Sun Yat-sen University

| Please fill out this questionnaire carefully and truthfully. The data you provide will help us understand your clinical information and accurately analyze your samples. Your data will be kept strictly confidential. Thank you very much for your participation and cooperation! | | | | | |
| --- | --- | --- | --- | --- | --- |
| Name |  | Gender |  | Date of Birth |  |
| Ethnic Group |  | Telephone Number |  | | |
| Native Place |  | Current Address |  | | |
| Marital Status |  | Education Level |  | Occupation |  |
| Height/cm |  | Alcohol Consumption | □ Never □ Occasionally □ Always | | |
| Weight/kg |  | Smoking Habit | □ Never □ Occasionally □ Always | | |
| Do you have a history of allergies?  □ No □ Yes, please list the allergens. | | | | | |
| Have you taken antibiotics in the past 2 months? (e.g., **cillin, oxacin, cef, metronidazole, **conazole) | | | | | |
| □ No | □ Yes, Drug Name: , Time: | | | | |
| How many times have you had diarrhea in the past 3 months?  □ None □ 1-3 □ >3 □ Chronic Diarrhea | | | | | |
| Do you have the following diseases? (Multiple choices)  □ None □ Hypertension □ Diabetes □ Hyperlipidemia □ Hyperuricemia □ Coronary Heart Disease □ Liver Cirrhosis □ Crohn's Disease □ Ulcerative Colitis □ Irritable Bowel Syndrome □ Depression □ Anxiety Disorder □ Other chronic diseases, please list. | | | | | |
| Have you had the following symptoms in the past 3 months? (Multiple choices)  □ None □ Hypertension □ Diabetes □ Hyperlipidemia □ Hyperuricemia □ Coronary Heart Disease □ Liver Cirrhosis □ Crohn's Disease □ Ulcerative Colitis □ Irritable Bowel Syndrome □ Depression □ Anxiety Disorder □ Other chronic diseases, please list. | | | | | |
| What is the shape of your stools? (Multiple choices) □ Separate hard lumps □ Lumpy and sausage - shaped □ Sausage - shaped with cracks on the surface  □ Like a smooth and soft sausage □ Soft, well - defined spots □ Pasty, rough - edged □ Liquid, shapeless | | | | | |
| Which of the following best describes your eating habits? □ Irregular diet □ Picky eating □ Often eat out □ Regular diet | | | | | |
| How often do you eat fresh fruits and vegetables? □ Every day □ At least once a week □ Rarely eat | | | | | |
| Do you often take probiotics? □ Yes □ No | | | | | |
| Do you often get impatient and irritable? □ Yes □ No | | | | | |
| What is your usual work and rest habit? □ Regular schedule □ Occasionally stay up late □ Often stay up late □ Work at night and sleep during the day | | | | | |
| What is your exercise habit? □ Exercise more than 3 times a week □ Exercise 1 - 3 times a week □ Do not exercise but have heavy physical labor □ Do not exercise and have light physical labor | | | | | |

| Please carefully recall the situation of edema and answer the following questions: | | |
| --- | --- | --- |
| At what age did you first experience edema?  At what age were you diagnosed with HAE? (□ Not yet diagnosed) How were you diagnosed with HAE? □ C1-INH testing □ Genetic testing □ Neither  What type of HAE do you have? □ HAE-1 □ HAE-2 □ HAE-nC1-INH □ Unclear | | |
| Since the onset of edema, have you ever experienced edema in the following areas? (Multiple choices) Skin: □ Face □ Eyelids □ Lips □ Tongue □ Neck □ Trunk □ Upper Limbs □ Lower Limbs □ Genital Tract Digestive System: □ Abdominal Pain □ Nausea □ Vomiting □ Difficulty Swallowing □ Abdominal Distension □ Diarrhea □ Ascites □ Constipation Respiratory System: □ Laryngeal Edema □ Difficulty Breathing □ Voice Change Urinary System: □ Difficulty Urinating □ Frequent Urination □ Urgency □ Painful Urination Nervous System: □ Headache Accompanying Diseases: □ Joint Swelling and Pain □ Vitiligo □ Psoriasis □ Thyroiditis □ Nephritis □ Viral Hepatitis □ Liver Cirrhosis □ Systemic Lupus Erythematosus □ Sjogren's Syndrome □ Oral Ulcers □ Others, please specify. | | |
| Age of Edema Onset | Age of first edema | Age group with frequent edema |
| Skin edema (facial, limb, etc.) |  |  |
| Gastrointestinal edema (abdominal pain, vomiting, etc.) |  |  |
| Throat edema (difficulty breathing, changes in voice, etc.) |  |  |
| What is your most common symptom since the onset of edema? □ Occasional limb swelling with no other symptoms □ The main symptom is skin edema, and gastrointestinal edema (abdominal pain, vomiting, etc.) and laryngeal edema are rare □ The main symptom is gastrointestinal edema, and skin edema and laryngeal edema are relatively rare □ The main symptom is laryngeal edema, accompanied by intermittent abdominal pain and skin swelling □ Skin edema, gastrointestinal edema, and laryngeal edema are all relatively common □ None of the above, the most common manifestation is. | | |
| Which of the following factors trigger your edema attacks? (Multiple choices) □ Emotional fluctuations □ Hormonal changes □ Weather changes □ Dust □ Food □ Medications □ Alcohol □ Non - alcoholic beverages □ Exercise □ Lack of sleep □ Fatigue □ Infection □ Trauma □ Surgery □ Dental treatment □ Blood donation □ Constipation □ Diarrhea □ No triggering factors □ Others: | | |
| Are there any prodromal symptoms before the onset of skin edema (facial, limb, etc.)? (such as wind clusters, red spots, etc.)  □ Nothing □ Yes, for example  Are there any prodromal symptoms before the onset of gastrointestinal edema (abdominal pain, vomiting, etc.)? (such as wind clusters, red spots, etc.)  □ Nothing □ Yes, for example  Are there any prodromal symptoms before the onset of laryngeal edema (difficulty breathing, changes in voice, etc.)? (such as wind clusters, red spots, etc.)  □ Nothing □ Yes, for example | | |
| Untreated duration of edema | Start relieving  (such as hours, days, etc.) | complete remission  (such as hours, days, etc.) |
| Skin edema (facial, limb, etc.) |  |  |
| Gastrointestinal edema (abdominal pain, vomiting, etc.) |  |  |
| Laryngeal edema (difficulty breathing, changes in voice, etc.) |  |  |
| Is there a correlation between skin edema, gastrointestinal edema (abdominal pain, vomiting, etc.), and laryngeal edema attacks?  □ No, the occurrence of the three is unrelated  □ Related, the order of edema occurrence is: skin edema → gastrointestinal edema → laryngeal edema  □ Related, the order of edema occurrence is: skin edema → laryngeal edema → gastrointestinal edema  □ Related, the order of edema occurrence is: gastrointestinal edema → skin edema → laryngeal edema  □ Related, the order of edema occurrence is: gastrointestinal edema → laryngeal edema → skin edema  □ None of the above. The order of edema occurrence is: | | |
| Is there a correlation between the relief of skin edema, gastrointestinal edema (abdominal pain, vomiting, etc.), and laryngeal edema?  □ No, the relief of the three is not related  □ Related, the order of edema relief is: skin edema → gastrointestinal edema → laryngeal edema  □ Related, the order of edema relief is: skin edema → laryngeal edema → gastrointestinal edema  □ Related, the order of edema relief is: gastrointestinal edema → skin edema → laryngeal edema  □ Related, the order of edema relief is: gastrointestinal edema → laryngeal edema → skin edema  □ None of the above. The order of edema relief is: | | |
| How does the frequency of edema attacks vary with age?  □ As age increases, there are more and more episodes of edema  □ As age increases, the occurrence of edema decreases  □ There is little relationship between age and frequency of edema attacks | | |
| Have you ever been diagnosed with the following diseases since the onset of edema? (Multiple Choice)  □ Urticaria □ Urticaria vasculitis □ Allergic reactions □ Asthma  □ Acute appendicitis □ Acute gastroenteritis □ Acute pancreatitis □ Irritable bowel syndrome  □ Neurosis □ Anxiety disorder □ Depression  □ Other: | | |
| Have you undergone endotracheal intubation due to laryngeal edema since the onset of edema?  □ No □ Yes, in total times | | |
| Have you undergone tracheotomy due to laryngeal edema since the onset of edema?  □ No □ Yes, in total times | | |
| Have you undergone abdominal surgery for unexplained abdominal pain since the onset of edema?  □ No □ Yes, in total times, the surgeries are | | |
| What methods do you usually use to alleviate the symptoms of edema? (Multiple Choice)  □ Ice or cold compress  □ Medication relief (over-the-counter or prescription drugs), such as  □ Avoid triggering factors (such as specific foods, medications, etc.) other: | | |

| Please carefully recall the suspected illness in your family and answer the following questions: |
| --- |
| Are you the first person in your family to be diagnosed with HAE?  □ yes □ No, the first person in our family to be diagnosed with HAE was mine |
| Have your parents experienced edema or been diagnosed with HAE?  □ No □ Yes  My has experienced edema, mainly manifested as □ skin edema □ gastrointestinal edema (abdominal pain, etc.) □ laryngeal edema  My has experienced edema, mainly manifested as □ skin edema □ gastrointestinal edema (abdominal pain, etc.) □ laryngeal edema  My has diagnosed with HAE |
| Has your child experienced edema or been diagnosed with HAE?  □ No □ Yes  I have son and daughter  My has experienced edema, mainly manifested as □ skin edema □ gastrointestinal edema (abdominal pain, etc.) □ laryngeal edema  My has experienced edema, mainly manifested as □ skin edema □ gastrointestinal edema (abdominal pain, etc.) □ laryngeal edema  My has experienced edema, mainly manifested as □ skin edema □ gastrointestinal edema (abdominal pain, etc.) □ laryngeal edema  My has diagnosed with HAE |
| Has your brother or sister ever had edema or been diagnosed with HAE?  □ No □ Yes  I have brother and sister and sister  My has experienced edema, mainly manifested as □ skin edema □ gastrointestinal edema (abdominal pain, etc.) □ laryngeal edema  My has experienced edema, mainly manifested as □ skin edema □ gastrointestinal edema (abdominal pain, etc.) □ laryngeal edema  My has experienced edema, mainly manifested as □ skin edema □ gastrointestinal edema (abdominal pain, etc.) □ laryngeal edema  My has diagnosed with HAE |
| Which other relatives in your family (including non direct relatives) have experienced edema or been diagnosed with HAE?  Edema has occurred:  Diagnosed with HAE: |

| Please carefully recall the medication and treatment situation, and answer the following questions: | | | | |
| --- | --- | --- | --- | --- |
| Have you ever used danazol?  □ No,  □ Yes, the first time I used it was , dosage and usage are ,  totally used (Years/Months), the last oral administration time was | | | | |
| Do you think danazol can effectively control edema attacks? (If not used, it can be skipped)  □ Can be controlled □ Partial control □ Cannot be controlled | | | | |
|  | Frequency of attacks (average per year) | | Severity (0 without swelling, 1-3 mild, 4-7 moderate, 8-10 severe)(Please choose an integer between 0-10) | |
|  | Before treatment | After treatment | Before treatment | After treatment |
| Skin edema |  |  |  |  |
| Gastrointestinal edema (abdominal pain, etc.) |  |  |  |  |
| Laryngeal edema |  |  |  |  |
| What are the side effects of taking danazol? (Multiple Choice) (If not used before, can be skipped)  □ No obvious side effects □ Abnormal liver function □ Menstrual disorders □ Acne □ Weight gain  □ Bone and joint pain □ Breast atrophy □ The sound becomes louder □ Increased hair growth  □ Increased skin oil content □ hematuria □ Nasal bleeding □ Gingival bleeding □ Cataract  □ Headache □ Decreased vision □ diplopia □ Vomiting □ Leukocytosis  □ Acute pancreatitis □ Multiple neuritis □ other: | | | | |
| Have you ever received an injection of lanadelumab?  □ No  □ Yes, the first time I used it was year Month, dosage and usage are , totally used (Year/Month), the last injection time was year month day | | | | |
| Do you think that lanadelumab can effectively control edema attacks? (If not used, it can be skipped)  □ Can be controlled □ Partial control □ Can not be controlled | | | | |
|  | Frequency of attacks (average per year) | | Severity (0 without swelling, 1-3 mild, 4-7 moderate, 8-10 severe)(Please choose an integer between 0-10) | |
|  | Before treatment | After treatment | Before treatment | After treatment |
| Skin edema |  |  |  |  |
| Gastrointestinal edema (abdominal pain, etc.) |  |  |  |  |
| Laryngeal edema |  |  |  |  |
| What are the side effects of injecting lanadelumab? (Multiple Choice) (If not used before, can be skipped)  □ No obvious side effects  □ Various reactions at the injection site (including pain, erythema, bruising, hematoma, swelling, induration, rash, itching, fever, etc.)  □ Allergic reactions (including rash, itching, shortness of breath, difficulty breathing, decreased blood pressure, persistent abdominal pain, vomiting in other parts of the body)  □ upper respiratory tract infection □ Headache Muscle pain □ Dizziness and headache □ Diarrhea □ Elevated transaminase levels  □ other: | | | | |
| Have you ever received an injection of icatibant?  □ No  □ Yes, the first time I used it was year Month, dosage and usage are , totally used (Year/Month), the last injection time was year month day | | | | |
| Do you think icatibant can effectively control edema attacks? (If not used, it can be skipped)  □ Skin edema can be controlled. Inject approximately when edema occurs, relief begins after minutes/hour, Complete relief after  hours; □ Skin edema can not be controlled.  □ Gastrointestinal edema can be controlled. Inject approximately when edema occurs, relief begins after minutes/hour, Complete  relief after hours; □ Gastrointestinal edema can not be controlled.  □ Laryngeal edema can be controlled. Inject approximately when edema occurs, relief begins after minutes/hour, Complete  relief after hours; □ Laryngeal edema can not be controlled. | | | | |
| What are the side effects of your injection of icatibant ? (Multiple Choice) (If not used before, can be skipped)  □ No obvious side effects  □ Various reactions at the injection site (including pain, erythema, bruising, hematoma, swelling, induration, rash, itching, fever, etc.)  □ Allergic reactions (including rash, itching, shortness of breath, difficulty breathing, decreased blood pressure, persistent abdominal pain, vomiting in other parts of the body)  □ Dizziness and headache □ Nausea and vomiting □ Rash, erythema, itching, urticaria □ Elevated transaminase levels  □ other: | | | | |
| Have you used any other medications to treat HAE besides this?  □ No □ Yes, for example(medicine) | | | | |
| Do you often take certain medications due to other illnesses?  □ No □ Yes, I often suffer from (illness) taking (medication)  This medication has an impact on HAE, What is the impact?  □ No impact □ Relieve the condition □ Aggravate the condition | | | | |

| Please carefully recall the swelling situation in the past month and answer the following questions: | | | |
| --- | --- | --- | --- |
| Onset of skin edema | seconds | Seek emergency medical attention due to skin edema | seconds |
| The severity of skin edema (0 without swelling, 1- 3 mild,  4-7 moderate, 8-10 severe) | | (Please choose an integer between 0-10) | |
| Emergency measures for skin edema | |  | |
| Onset of laryngeal edema | seconds | Seek emergency medical attention due to laryngeal edema | seconds |
| The severity of laryngeal edema (0 without swelling, 1-3 mild,  4-7 moderate, 8-10 severe) | | (Please choose an integer between 0-10) | |
| Emergency measures for laryngeal edema (abdominal pain, etc.) | |  | |
| Onset of gastrointestinal edema (abdominal pain, etc.) | seconds | Seek emergency medical attention due to gastrointestinal edema (abdominal pain, etc.) | seconds |
| The severity of gastrointestinal edema (abdominal pain, etc.) (0 without swelling,  1-3 mild, 4-7 moderate, 8-10 severe) | | (Please choose an integer between 0-10) | |
| Emergency measures for gastrointestinal edema (abdominal pain, etc.) | |  | |
| From edema to complete disappearance of edema | | Usually hours, shortest hours, longest hours | |
| Have you taken danazol in the past month | | □ No □ Yes, the dosage is | |
| Have you received any injections of lanadelumab in the past month | | □ No □ Yes, the dosage is  The frequency is The time is | |
| Have you received any injections of icatibant in the past month | | □ No □ Yes, the dosage is  The frequency is The time is  effect: minutes/hours can alleviate | |
| The impact of edema on your life over the past month | | □ No episodes occurred  □ Discomfort, but does not affect daily life  □ Affects daily life  □ Unable to work or study, seeking treatment is necessary | |

| Please carefully recall the swelling situation in the past year and answer the following questions: | | | |
| --- | --- | --- | --- |
| Onset of skin edema | seconds | Seek emergency medical attention due to skin edema | seconds |
| The severity of skin edema (0 without swelling, 1- 3 mild,  4-7 moderate, 8-10 severe) | | (Please choose an integer between 0-10) | |
| Emergency measures for skin edema | |  | |
| Onset of laryngeal edema | seconds | Seek emergency medical attention due to laryngeal edema | seconds |
| The severity of laryngeal edema (0 without swelling, 1-3 mild,  4-7 moderate, 8-10 severe) | | (Please choose an integer between 0-10) | |
| Emergency measures for laryngeal edema (abdominal pain, etc.) | |  | |
| Onset of gastrointestinal edema (abdominal pain, etc.) | seconds | Seek emergency medical attention due to gastrointestinal edema (abdominal pain, etc.) | seconds |
| The severity of gastrointestinal edema (abdominal pain, etc.) (0 without swelling,  1-3 mild, 4-7 moderate, 8-10 severe) | | (Please choose an integer between 0-10) | |
| Emergency measures for gastrointestinal edema (abdominal pain, etc.) | |  | |
| From edema to complete disappearance of edema | | Usually hours, shortest hours, longest hours | |
| Have you taken danazol in the past year | | □ No □ Yes, the dosage is | |
| Have you received any injections of lanadelumab in the past year | | □ No □ Yes, the dosage is  The frequency is The time is | |
| Have you received any injections of icatibant in the past year | | □ No □ Yes, the dosage is  The frequency is The time is  effect: minutes/hours can alleviate | |
| The impact of edema on your life over the past year | | □ No episodes occurred  □ Discomfort, but does not affect daily life  □ Affects daily life  □ Unable to work or study, seeking treatment is necessary | |
