# Supplementary material for: Clinical features of hereditary angioedema involving the gastrointestinal tract: A retrospective analysis
Source: World Allergy Organ J. 2026 Jan 31;19(2):101252. doi: 10.1016/j.waojou.2026.101252 (PMC12886538; doi:10.1016/j.waojou.2026.101252)
Supplement: Multimedia component 3 [file mmc3.doc]

**Supplementary figure and tables**


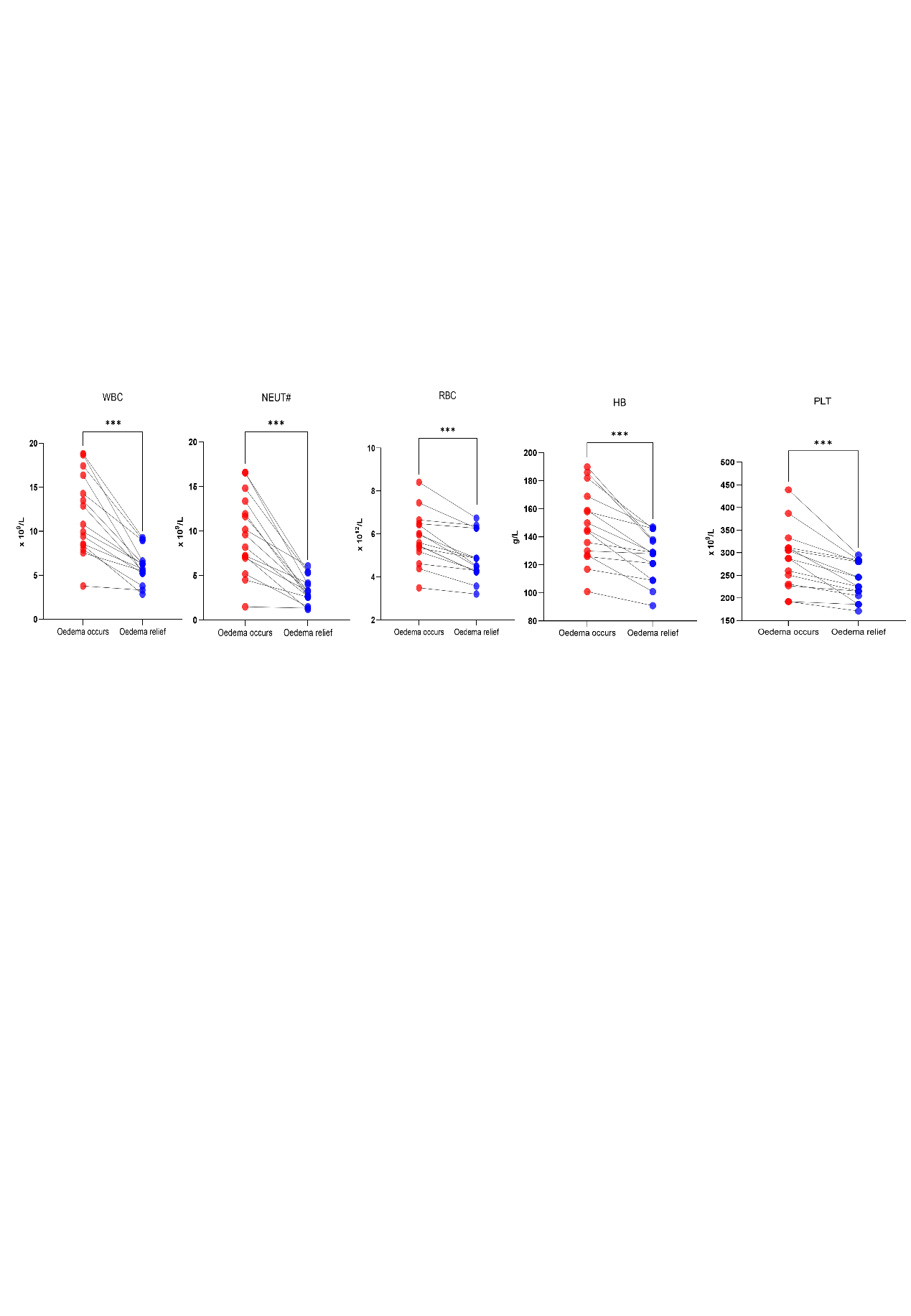


Supplementary Fig. 1: Comparison of different reactant levels between the phases of edema attacks and remission (n=15).

Supplementary Table. 1: The comorbidities of gastrointestinal edema (n=53)

| Comorbidity（N=53） | n（%） |
| --- | --- |
| Metabolic diseases | 15（28.3） |
| Chronic inflammatory diseases | 11（20.8） |
| Joint swelling and pain | 9（17.0） |
| Recurrent oral ulcers | 7（13.2） |
| Psychological disorders | 4（7.5） |
| Sjögren's syndrome | 3（5.7） |
| Neoplastic diseases | 2（3.8） |

Supplementary Table. 2: The proportion of patients with different triggers in gastrointestinal edema attacks (n=53) (Hormonal fluctuations including puberty, menstrual cycle, pregnancy, childbirth, and other physiological changes)

| Triggers（N=53） | n（%） |
| --- | --- |
| Physical exhaustion | 27（50.9） |
| Mood swings | 27（50.9） |
| Weather changes | 21（39.6） |
| Dietary factors | 20（37.7） |
| External trauma | 13（24.5） |
| Hormonal fluctuations | 12（22.6） |
| Sleep deprivation | 11（20.8） |
| Physical activity | 11（20.8） |
| Spontaneity | 9（17.0） |
| Alcohol | 8（15.1） |
| Infection | 7（13.2） |
| Drug | 6（11.3） |
| Surgery | 3（5.7） |
| Non-alcoholic beverages | 2（3.8） |
| Exposure to chemicals | 2（3.8） |
